# Supplementary material for: Three-dimensional label-free imaging and quantification of lipid droplets in live hepatocytes
Source: Sci Rep. 2016 Nov 22;6:36815. doi: 10.1038/srep36815 (PMC5118789; doi:10.1038/srep36815)
Supplement: Supplementary Information [file srep36815-s5.pdf]

Supplementary Information

Three-dimensional label-free imaging and quantification of lipid droplets in live hepatocytes

Kyoohyun Kim[1], SeoEun Lee[1,†], Jonghee Yoon[1], JiHan Heo[1, ‡], Chulhee Choi[2], and YongKeun Park[1,3,*]

[1]Department of Physics, Korea Advanced Institute of Science and Technology, Daejeon 34141, Republic of Korea.

[2]Department of Bio and Brain Engineering, Korea Advanced Institute of Science and Technology, Daejeon 34141, Republic of Korea.

[3]TOMOCUBE, Inc., Daejeon 34051, Republic of Korea

[†]Present address: Graduate School of Arts and Sciences, Columbia University, NY 10032, USA

[‡]Present address: College of Medicine, Seoul National University, Seoul 03080, Republic of Korea

*Correspondence:

Prof. YongKeun Park,

E-mail: yk.park@kaist.ac.kr,

Tel: +82-42-350-2514, Fax: +82-42-350-2510

Descriptions for supplementary movies

Supplementary movies:

Supplementary Movie 1
The 3-D rendered isosurface image of 3-D RI distribution of a Huh-7 cell from various viewing angles.

Supplementary Movie 2
The 3-D rendered isosurface image of 3-D RI distribution of an untreated Huh-7 cell from various viewing angles.

Supplementary Movie 3
The 3-D rendered isosurface image of 3-D RI distribution of an oleic acid treated Huh-7 cell from various viewing angles.

Supplementary Movie 4
The time-lapse 3-D rendered isosurface images of 3-D RI distribution of a hepatocyte. Each frame of 3-D RI distribution was measured every 3 seconds for 93 seconds.

Supplementary Figure 1
The distribution of RI contrast values in the background region.

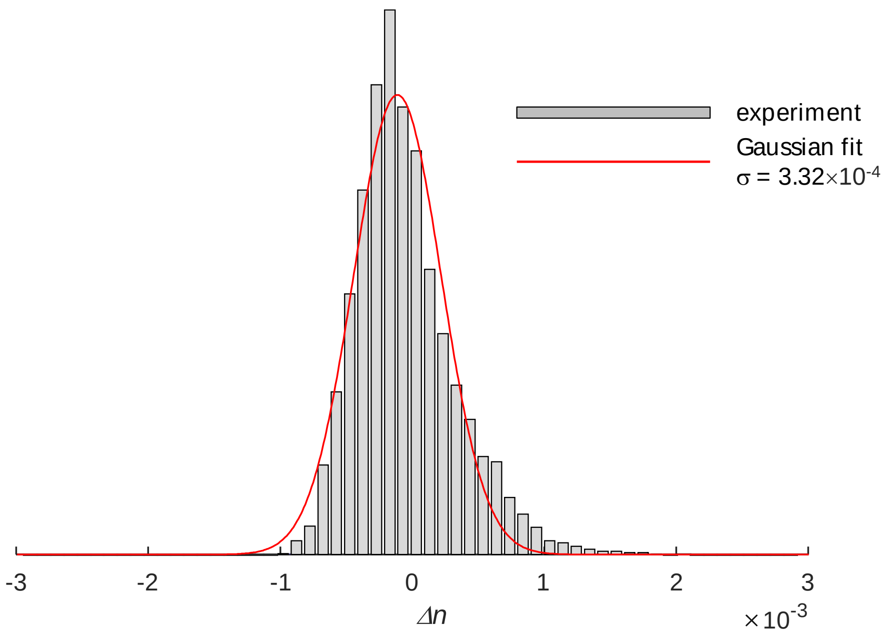

Supplementary Figure 1. The distribution of RI contrast values in the background region. The solid red line indicates a Gaussian fit with the standard deviation of 3.32×10^{-4}.
